# Taxonomic and Life History Bias in Herbicide Resistant Weeds: Implications for Deployment of Resistant Crops

**DOI:** 10.1371/journal.pone.0071916

**Published:** 2013-09-09

**Authors:** Jodie S. Holt, Shana R. Welles, Katia Silvera, Ian M. Heap, Sylvia M. Heredia, Alejandra Martinez-Berdeja, Kai T. Palenscar, Lynn C. Sweet, Norman C. Ellstrand

**Affiliations:** 1 Department of Botany and Plant Sciences, University of California Riverside, Riverside, California, United States of America; 2 Smithsonian Tropical Research Institute, Balboa, Ancon, Republic of Panama; 3 International Survey of Herbicide Resistant Weeds, Corvallis, Oregon, United States of America; Virginia Tech, United States of America

## Abstract

Evolved herbicide resistance (EHR) is an important agronomic problem and consequently a food security problem, as it jeopardizes herbicide effectiveness and increases the difficulty and cost of weed management. EHR in weeds was first reported in 1970 and the number of cases has accelerated dramatically over the last two decades. Despite 40 years of research on EHR, why some weeds evolve resistance and others do not is poorly understood. Here we ask whether weed species that have EHR are different from weeds in general. Comparing taxonomic and life history traits of weeds with EHR to a control group (“the world's worst weeds”), we found weeds with EHR significantly over-represented in certain plant families and having certain life history biases. In particular, resistance is overrepresented in Amaranthaceae, Brassicaceae and Poaceae relative to all weeds, and annuality is ca. 1.5 times as frequent in weeds with EHR as in the control group. Also, for perennial EHR weeds, vegetative reproduction is only 60% as frequent as in the control group. We found the same trends for subsets of weeds with EHR to acetolactate synthase (ALS), photosystem II (PSII), and 5-enolpyruvylshikimate-3-phosphate (EPSP) synthase-inhibitor herbicides and with multiple resistance. As herbicide resistant crops (transgenic or not) are increasingly deployed in developing countries, the problems of EHR could increase in those countries as it has in the USA if the selecting herbicides are heavily applied and appropriate management strategies are not employed. Given our analysis, we make some predictions about additional species that might evolve resistance.

## Introduction

Evolved herbicide resistance (EHR) has become a threat to agriculture and consequently a food security problem worldwide [1], [2]. EHR in weeds was first reported in 1970 [3], [4] and widely studied in the 1970s through 1990s [3]. The number of cases has accelerated dramatically over the last two decades [5]. The evolution of resistance to multiple herbicides with different modes of action has also been found within numerous weed species to date [6]. The discovery of resistance to glyphosate (the dominant herbicide worldwide) in the 1990s [7], the introduction of transgenic glyphosate-resistant crops in 1996 [8], and the recent expansion of cases of evolved resistance to glyphosate in weeds, likely due to greater glyphosate usage, have inspired a renewal of interest and resurgence of research into this phenomenon.

Despite 40 years of research on EHR, it is not clear why some weeds evolve resistance faster than others. Baker's list of characteristics that might be expected in the “ideal weed” is well known [9]; one might expect weeds with EHR to possess a subset of these traits. A cursory review of available data in 2001 revealed that some weeds have a greater propensity to evolve resistance than others [10]. This observation was attributed to opportunity, as many resistant weeds are among the world's worst weeds [11], [12], are widespread, and occur in many cropping systems [10]. Well before EHR was discovered, heritable variability, breeding system, reproductive capacity, annuality, and population size were predicted to correlate with evolution of herbicide resistance [13]. Other plant factors can affect the evolution of resistance, including mutation frequency, generation time, fitness in absence of the herbicide, plasticity, and soil seed reservoir [14], as well as mode of inheritance of resistance, population size, seed dormancy, and gene flow by pollen and seed [15]. While these factors have been tested in models predicting evolution of resistance [14], [16], few have been tested empirically.

Given that taxonomic families are relatively cohesive internally but generally vary from one another in ecological traits, we might expect EHR to be represented nonrandomly among plant families. Similarly, based on observations and reports of the propensity for resistance to evolve within certain genera or species, we would expect weeds with HER to be ecologically and taxonomically different than weeds in general. Here we ask whether weed species that have EHR are different from weeds in general and if evolution of resistance to multiple herbicides follows the same patterns.

## Materials and Methods

A list of species with evolved herbicide resistance EHR; (henceforth, the EHR list) was obtained from the database at the website “International Survey of Herbicide Resistant Weeds” http://www.weedscience.org/In.asp created and maintained by Ian Heap with support from the Herbicide Resistance Action Committee, the North American Herbicide Resistance Action Committee, and the Weed Science Society of America [5]. At the time of our study (May 2012), the inventory included 187 species in 31 plant families. For statistical comparison, we chose the two volumes by Holm et al. that inventory the “World's Worst Weeds” and “World Weeds” [11], [12] (henceforth the control list) including 201 species in 49 plant families. Entries from different subspecies of the same species were lumped together. The taxonomy for both lists was updated using the USDA ARS Germplasm Resources Information Network website “GRIN Taxonomy for Plants”, http://www.ars-grin.gov/cgi-bin/npgs/html/index.pl?language=en [17]. All scientific names were then checked against the “The Plant List” website database, http://www.theplantlist.org/, which was also used to obtain current plant family assignments. The two updated lists are presented in Table S1.

Information regarding plant life history was extracted from the Holm et al. [11], [12] publications, if available. For species not present in those publications, the information was obtained from major floras (e.g. [18], [19],) or citations for individual species entries in the USDA ARS “GRIN Taxonomy for Plants” [17].

All comparisons between the EHR list and the control list were done using a Parson's chi-square statistic (2-tailed test) with one degree of freedom; α = 0.05 was used to determine significance. This test was used because it is appropriate for comparisons between theoretical and experimental populations where a data set is large and observations are independent. Calculations were done using R statistical package [20].

We constructed a phylogenetic tree hypothesis for the relationship among plant families with EHR using Phylomatic 2 and the Angiosperm Phylogeny Group 3 derived megatree (http://www.phylodiversity.net/phylomatic/phylomatic.html). Presence and absence of EHR to acetolactate synthase (ALS)- and photosystem II (PSII)-inhibitors were mapped onto the same tree. Character state reconstruction for each EHR group was performed by maximum likelihood using a marginal probability reconstruction with the Asymmetrical parameter Markov-k model of evolution in Mesquite version 2.74 [21]. The results are displayed as likelihood states reported as proportion of the total likelihood and represented as pie charts in each branch node within the tree.

## Results and Discussion

Comparisons between the control and EHR lists showed sixty-two species in 19 families are found in common on both lists, representing 33% of the 187 species on the EHR list and 31% of the 201 species on the control list. When ranked by number of species, the same plant families were dominant on both lists (Table 1). Poaceae, Asteraceae, Amaranthaceae, and Brassicaceae were among the top six families on each list, comprising 75% and 57% of species on the EHR and control lists, respectively. This is not surprising since plant families that are overrepresented in the global weed flora would have a greater probability of herbicide exposure and selection of species with EHR [10].

**Table 1:** Rank of plant families by number and % of species in control and EHR lists.

	Control list^1^			EHR List		
Rank	Plant Family	Species		Plant Family	Species	
		(no.)	(%)		(no.)	(%)
**1**	Poaceae	47	23	Poaceae	60	32
**2**	Asteraceae	30	15	Asteraceae	33	18
**3**	Cyperaceae	12	6	Amaranthaceae	18	10
**4**	Amaranthaceae	10	5	Brassicaceae	17	9
**5**	Polygonaceae	8	4	Alismataceae	6	3
**6**	Brassicaceae	7	4	Polygonaceae	6	3
	Total	114	57	Total	140	75

1 Control list includes 201 species from “World's Worst Weeds of Holm et al. (1977, 1979); Evolved herbicide resistance (EHR) list includes 187 species from the website http://www.weedscience.org/In.asp.

Despite the taxonomic overlap between the EHR and control lists at the family level, we found significant differences with regard to species abundance within families (Table 2). In particular, EHR was overrepresented relative to the control in three of the four aforementioned families (Poaceae, Amaranthaceae, and Brassicaceae but not Asteraceae) (Table 2). While all but three families on the EHR list were also on the control list, 21 plant families that had one or more species on the control list were lacking altogether on the EHR list.

**Table 2:** Comparison of plant family and life history traits.

		All Resistance				
		Control list (%)^1^	Complete EHR list (%)^1^	χ^2^ statistic	*p*-value	Difference from control list^2^
**Family**	Amaranthaceae	5	10	8.55	0.004	+
	Brassicaceae	4	9	17.55	2.87×10^−5^	+
	Poaceae	23	32	7.91	0.0049	+
**Life-history traits**	Annuality^3^	53	86	64.26	1.08×10^−15^	+
	Perenniality	43	11	64.32	1.06×10^−15^	−
	Vegetative Reproduction^4^	68	41	18.11	2.08×10^−5^	−

		ALS Resistance				
		Control list (%)	EHR to ALS- inhibitors (%)	χ^2^ statistic	*p*-value	Difference from control list
**Family**	Amaranthaceae	5	11	7.88	0.005	+
	Brassicaceae	4	15	40.1	2.4×10^−8^	+
	Poaceae	23	23	0.013	NS	0
**Life-history traits**	Annuality	53	87	35.18	3.08×10^−9^	+
	Perenniality	43	9	39	4.23×10^−10^	−
	Vegetative Reproduction	68	48	4.91	0.03	−

		PSII Resistance				
		Control list (%)	EHR to PSII- inhibitors (%)	χ^2^ statistic	*p*-value	Difference from control list
**Family**	Amaranthaceae	5	24	51.8	6.06×10^−13^	+
	Brassicaceae	4	6	1.3	NS	0
	Poaceae	23	30	2.21	NS	0
**Life-history traits**	Annuality	53	97	35.41	2.7×10^−9^	+
	Perenniality	43	2	43.34	4.59×10^−10^	−
	Vegetative Reproduction	68	25	6.6	0.01	−

		Glycine Resistance				
		Control list (%)	EHR to glycine herbicides (%)	χ^2^ statistic	*p*-value	Difference from control list
**Family**	Amarthaceae	5	14	3.85	0.049	+
	Brassicaceae	4	0			None with glycine resistance
	Poaceae	23	48	6.88	0.0087	+
**Life-history traits**	Annuality	53	176	3.73	0.05	+
	Perenniality	43	17	4.95	0.026	−
	Vegetative Reproduction	68	43			Not enough data

		Multiple Resistance				
		Control list (%)	Multiple resistance list (%)	χ^2^ statistic	*p*-value	Difference from control list
**Family**	Amaranthaceae	5	13	4.79	0.0286	+
	Brassicaceae	4	8	1.92	0.1658	+
	Poaceae	23	48	12.99	0.000314	+
**Life-history traits**	Annuality	53	94	20.38	6.35×10^−6^	+
	Perenniality	43	3	19.66	9.26×10^−6^	−
	Vegetative Reproduction	68	67			Not enough data

Plant family representation and life history traits in complete EHR list and in lists of weeds with EHR to ALS-inhibitors, PSII-inhibitors, glycine herbicides, and with multiple herbicide resistance. Pearson's chi-square statistic (2-tailed test) with 1 degree of freedom was used to assess the difference between EHR lists and the control list. An α = .05 was used to determine significance. Calculations were done using R statistical package [20]).

1 Control list, n = 201; complete EHR list, n = 187; EHR to ALS inhibitors, n = 99; EHR to PSII inhibitors, n = 66; EHR to glycine herbicides, n = 21; multiple resistance, n = 40.

2 +, EHR is over-represented in the family or life history trait; −, EHR is under-represented in the family or life history trait; 0, no difference between control and EHR list.

3 Calculation of % annuality excluded species that could also be biennial or perennial; calculation of % perenniality excluded species that could also be annual or biennial.

4 Vegetative reproduction is a % of non-annuals.

An early attempt to relate EHR to phylogeny revealed broad trends at the level of the superorder [22]. EHR was not found in woody tropical superorders, rather was found in groups predominantly associated with temperate agriculture, likely reflecting the areas of greatest herbicide use. Perhaps because fewer cases of EHR were known at that time, no phylogenetic trends in EHR were evident, in contrast to our results.

Because the EHR list is heterogeneous with evolved resistance to a variety of herbicide groups, we also asked whether the same taxonomic bias was present for the two most prevalent types of EHR, that is, to ALS- and PSII-inhibitor herbicide groups, as well as for resistance to the world's most important herbicide, glyphosate [8]. For some families overrepresentation of resistance to the three herbicide groups was present. Amaranthaceae were overrepresented in all three herbicide groups, Brassicaceae were overrepresented in EHR to ALS-inhibitors, and Poaceae were overrepresented in EHR to glyphosate, when the EHR list for each of these herbicide groups was compared to the control list (Table 2). Characteristics of these herbicides could partially account for results in some instances. For example, many grasses are naturally resistant to ALS-inhibitors and therefore are less likely to be treated with herbicides from this group. so it is not surprising that Poaceae is not overrepresented in this herbicide group. While natural variation in resistance to herbicides could account for lack of representation by some taxa, it cannot account for overrepresentation of EHR in these families relative to weeds in general. These results suggest that there is a strong phylogenetic signal in these families for frequent selection of resistance to specific herbicide groups [14], [15].

**Figure 1:**
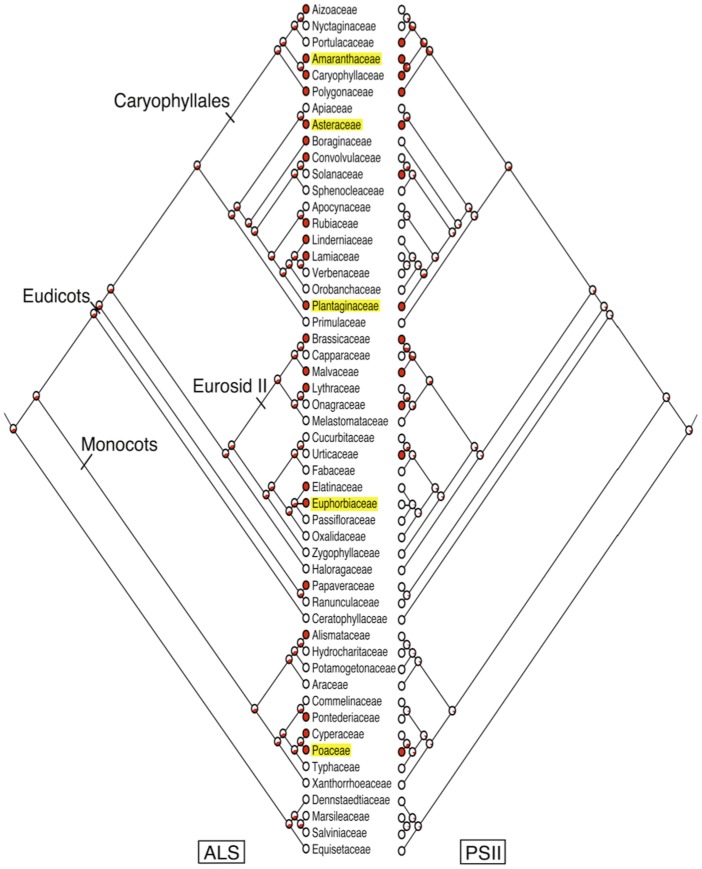
Phylogenetic tree showing the relationship among 52 plant families. EHR to ALS-inhibitors (left panel) and PS-II inhibitors (right panel) were mapped onto the tree using Mesquite version 2.74. Families with EHR to glyphosate are highlighted in yellow. Relevant plant lineages are labeled within the tree nodes. Lineages that show EHR are depicted in red. The red area within each pie chart indicates the relative support for different ancestor states.

We examined the phylogenetic relationship between EHR and each of these herbicide groups and found different trends (Figure 1). The breadth of phylogenetic distribution appears to correlate with the ease of selection for resistance to distinct herbicide groups. EHR to ALS-inhibitors is spread widely over plant families (Figure 1). This trend can be explained by the strong selection pressure exerted by ALS inhibitors as well as the relative ease with which plants evolve resistance to ALS inhibitors. Several point mutations can confer EHR to ALS-inhibitors, which is followed by rapid evolution because the encoded gene is nuclear inherited and easily transmitted by seed and pollen [23].

In contrast, although PSII inhibitors have long soil residual activity, selection of EHR to the PSII-inhibitor herbicide group is more difficult because in most cases reported it requires a specific point mutation in the psbA gene, which is generally maternally inherited [24]. Accordingly, our analysis reveals that evolved PSII-inhibitor resistance is much more phylogenetically restricted than that to ALS-inhibitors. Poaceae is the only monocot family that has EHR to PSII-inhibitors; in eudicot families, there is a notable concentration in the Caryophyllales and Eurosid II clades with the other cases present in only four weakly related eudicot families (Figure 1).

EHR to glyphosate is more complex, involving target and non-target site resistance mechanisms, changes in the translocation of glyphosate to meristematic regions [20], and virtually no soil residual activity. Although there is abundant intraspecific variability in glyphosate susceptibility in weeds, EHR to this herbicide was not discovered for two decades following its introduction [8]. The relatively slow appearance of EHR to glyphosate compared to other herbicide groups was initially attributed to genetic and biochemical constraints, restricted use, and lack of soil residual activity resulting in low selection pressure for resistance [8]. Indeed, EHR to glyphosate currently shows a very restrictive phylogenetic distribution with almost half of species found in a single family, Poaceae, and six of the eight genera with EHR in that family are in two closely related subfamilies, Panicoideae and Chloridoideae. Dramatic increases in glyphosate use where transgenic glyphosate-resistant crops are planted have intensified selection pressure for EHR, which might alter these phylogenetic patterns in the future.

Cumulatively, our results for the three herbicide groups tested suggest that as selection of resistance to an herbicide group becomes increasingly difficult, the taxonomic distribution of EHR will be subject to increasing phylogenetic constraint. Genetic variation for resistance appears not to be uncommon in weed populations, however, little is known about actual rates of mutation to EHR for any weed species [15]. More information about genetics of resistance to a particular herbicide (frequency, number, dominance, and fitness cost of resistance genes) as well as about natural mutation frequencies, would improve our ability to understand underlying causes of the phylogenetic patterns detected here.

We also compared the full EHR list and the control list with respect to plant habit (annuality, bienniality, perenniality). We found that annual weeds are 1.5 times as frequent, whereas perennial weeds are only 0.27 times as frequent in the EHR list as in the control list (Table 2). We attribute this difference to the fact that short-lived species are recognized to have a more rapid response to directional selection than long-lived species, leading to greater opportunity for evolution of EHR [14], [15]. If length of life cycle is inversely correlated with EHR, then we would predict that because vegetative reproduction effectively extends generation time, perennials with vegetative reproduction should be highly underrepresented among weeds with EHR. As expected, we found the proportion of vegetative reproduction in perennials with EHR to be only 0.6 times that in the control list. The same trends were found in the three herbicide groups evaluated with respect to plant habit and vegetative reproduction (Table 2).

While many traits are predicted to correlate with EHR [13], [14], [15], some (e.g., fitness, plasticity, soil seed reserve, population size, and gene flow) are strongly influenced by environment and difficult to include in a phylogenetic analysis. Other life history traits, e.g., outcrossing rate, spontaneous hybridization, fecundity, and seed size, might help explain phylogenetic patterns in EHR. Unfortunately, available data are insufficient to allow further quantitative assessment or meta-analysis of these traits for weeds with EHR compared to weeds in general.

A total of 139 species across 44 families on the control list had no reports of EHR. All of these species are found in countries for which reports of EHR occur [5]; thus, the absence of EHR cannot be explained by lack of herbicide exposure or EHR reporting. We note that aquatic and wetland species are significantly more numerous on the control list than the EHR list (χ^2^ = 11.69, df = 2, p = 0.00063) [9], [10]. Despite the fact that aquatic weeds are widespread, restricted herbicide use in wetland habitats (with the exception of rice production) apparently precludes selection for EHR [25]. For other species on the control list where no obvious factors account for the lack of EHR, it is likely that habitat, cropping system, or other agronomic factors influence the probability of exposure to herbicides, and thus, selection for EHR.

We also examined patterns between individuals with resistance to multiple herbicides in different functional groups (herbicides with different modes of action). We found that there is a significant increase in multiple resistance in the Amaranthaceae and Poaceae compared with the control list. The abundance of multiple resistance in these families is not surprising given that they are also a subset of the families that have high levels EHR. We also analyzed multiple resistance from the perspective of the herbicides involved. We found that the majority of herbicides have a similar proportion of species with resistance to that herbicide and species with multiple resistance. However, we found that 62% of species with glyphosate resistance have multiple resistance compared to the approximately 30% among most other herbicide families. It is possible that the cause of increased multiple resistances that include glyphosate is due to the fact that glyphosate is a newer herbicide and is often applied to areas that already have resistance to other herbicides. Therefore, there are increased opportunities for evolution of resistance to glyphosate on a background of resistance to other herbicides.

A major mechanism for multiple herbicide resistance in plants is metabolism by cytochrome P450 monooxygenases [6], [26], [27]. As these enzymes can metabolize herbicides with different modes of action, their existence in weeds with EHR could strongly influence phylogenetic analysis. Unfortunately, while much is known about the role of these enzymes in insecticide resistance [28], little is known about their existence and role in weeds [6], [27]. To date most of the reports of cytochrome P450 monooxygenases have been for grass weeds [6], but the limited number of species for which P450-based EHR is known precludes using this information in our analysis.

We expect that herbicide resistance will continue to evolve in regions with the strongest herbicide selection pressure, in particular, in major agricultural countries. For example, considering the case of glyphosate in 2010, the United States, Brazil, and Argentina were #1, #2, and #3 with respect to acreage of crops engineered for glyphosate resistance, collectively comprising more than 80% of the world's acreage, which comprised 134 million ha in 2009 [29], [30]. Presently, there are 112 known instances of EHR to glyphosate [5]; 86 of these were found in the United States, Brazil, and Argentina (more than 75% of the total). While we acknowledge that glyphosate is used for more than weed control in transgenic crops, the strong relationship between their acreage and EHR cannot be ignored.

Our analysis has immediate implications for developing countries that are accelerating or about to accelerate their use of herbicides. As reported by the National Research Council [1], without careful attention to herbicide resistance management (e.g., appropriate herbicide rotations in combination with non-chemical control methods), increased use of herbicides could bring about new instances of EHR [31]. Our results show that new instances of EHR are more likely to be found for annual weeds in the families that we identified as EHR prone. To take a specific example, the recent historic information on glyphosate use is easily available. In 2010, in terms of area planted to glyphosate resistant crops, Paraguay ranked fifth in the world with 2.6 million hectares of glyphosate resistant soybean [29]. That crop was first introduced to Paraguay in 2004 [29]. With six years of intense use, there are now two reports of glyphosate resistance in Paraguay, both for *Digitaria insularis* (sourgrass, once in 2006 and later in 2008). The situation is not trivial; the latter report indicates an increasing infestation on the order of hundreds of acres at over a dozen locations. We note that this species follows the phylogenetic trends our analysis has identified. Sourgrass is a member of the Poaceae, a family overrepresented for both EHR in general and glyphosate resistance in particular. With continued glyphosate use in Paraguay we would predict some of the following species to rapidly evolve resistance: *Echinochloa colona* and *Eleusine indica*, reported to have EHR to other herbicide groups in South America, and even more worrisome, *Lolium* spp. and *Sorghum halepense*, reported to have glyphosate resistance elsewhere in South America. As herbicide resistant crops (transgenic or not) are increasingly deployed in developing countries, the problems of EHR will likely spread to those countries [32].

Pesticide resistance was first discovered in insects, then fungi, then plants [33], yet this type of analysis of phylogenetic patterns has never been done except at the gene level (e.g. [34], [35], but see [22]). We have shown that weeds with EHR are far from a random sample of weedy plant species. Our analysis indicates that certain species in certain families, particularly those species with shorter life cycles, will rapidly evolve herbicide resistance under high selection pressure. Accordingly, local weed managers and scientists can anticipate this evolution and employ recommended herbicide resistance management techniques to delay evolution toward herbicide resistance and preserve the efficacy of a given herbicide [31]. Further analysis of other traits of EHR will refine such strategies.

## Supporting Information

Table S1 **Complete list of species and life history traits used in this study.**
(XLSX)
